# “I just had the feeling that the interval training is more beneficial”: young adults' subjective experiences of physical fitness and the role of training modes

**DOI:** 10.3389/fspor.2023.1115944

**Published:** 2023-05-15

**Authors:** Hannes Gropper, Jannika M. John, Gorden Sudeck, Ansgar Thiel

**Affiliations:** ^1^ Institute of Sports Science, Eberhard Karls University Tübingen, Tübingen, Germany; ^2^ Interfaculty Research Institute for Sport and Physical Activity, Eberhard Karls University Tübingen, Tübingen, Germany

**Keywords:** physical inactivity, physical fitness, subjective experience, high-intensity interval training (HIIT), moderate intensity continuous training (MICT)

## Abstract

**Objective:**

Compelling evidence has shown that high-intensity interval training (HIIT) is associated with substantial increases in physical fitness. However, little is known about whether and how individuals experience these adaptations over time. The purpose of this study is, therefore, to explore how physically inactive young adults subjectively experience physical fitness and its development as they start to exercise and how these experiences relate to different intensity domains (i.e., moderate and severe) as well as to training modes [i.e., HIIT and moderate intensity continuous training (MICT)] and their sequential administration (i.e., HIIT–MICT and MICT–HIIT).

**Methods:**

Thirty-one inactive participants completed a 15-week two-period sequential exercise intervention in which they first trained for six weeks in a HIIT or a MICT group and subsequently switched training modes. Interviews using the biographical mapping method were conducted at the end of the intervention to assess participants’ exercise- and fitness-related experiences over the past weeks. To assess experiential patterns, we conducted a reflexive thematic analysis.

**Results:**

We developed three themes that represent the temporal and processual character of starting to exercise after a prolonged period of inactivity: (1) Inactive young adults are not all the same when starting to exercise; (2) Developing physical fitness is a multi-faceted and individual experience; and (3) Feeling physically fit (or not) fosters large-scale effects.

**Conclusion:**

Our results show that, in retrospect, participants mostly deemed HIIT to be more effective than MICT. Our findings also emphasize that physical fitness is a complex and subjective experience that manifests in various ways over time. The idiosyncrasy of physical fitness experiences reiterates the necessity for individually tailored exercise prescriptions instead of one-size-fits-all approaches.

## 1. Introduction

Regular physical activity (PA) and exercise are associated with many potential biopsychosocial health benefits [e.g., (1–3)]. At the same time, global PA-levels are low (4, 5), oftentimes not meeting recommended minimum doses to achieve health-enhancing benefits. Such phenomena led exercise researchers and public health initiatives to inquire how the promotion of active lifestyles can be designed to be low threshold and motivate as many people as possible to engage in regular PA and exercise. In this regard, the physiological benefits of PA and exercise are often emphasized. In addition, we assume that people must also *experience* and *feel* those physiological benefits in order to encourage continued participation (6).

In recent years, one topic of debate in the discussion on PA and exercise promotion has been the question of whether high-intensity interval training (HIIT) may be a viable option for public health purposes, in contrast, for example, to more traditional moderate intensity continuous training (MICT) programs (7, 8). Compared to MICT, which is commonly prescribed within the moderate-intensity domain [i.e., below the first lactate threshold (9)] and with a constant load, HIIT is generally characterized by alternating periods of severe intensity [which link to a substantial increase in pulmonary oxygen uptake and are above the second lactate threshold (9, 10)] and periods of rest or low intensity (11, 12). In this regard, HIIT is considered to be relatively safe and requires lower time commitment due to a reduced exercise volume while potentially fostering similar or even higher physiological adaptations than MICT (e.g., 13–15)—at least “when compared on a matched-work basis” (12).

Exercise physiologists have regularly argued that both HIIT and MICT can promote cardiometabolic health and cardiorespiratory fitness, although findings comparing their effectiveness are partially equivocal. While some studies suggest that HIIT and MICT might elicit similar physical fitness adaptations (i.e., primarily VO_2max_) for various age groups and samples over time [e.g., (16–18)], most studies have shown that HIIT usually tends to be associated with greater VO_2max_ improvements in exercise training interventions that ranged from approximately three to twelve weeks [e.g., (19–24)]. Yet, caution is advised as different findings regarding physiological outcomes might be attributable to different prescribed exercise intensities, volumes, and durations as well as methodological and analytical approaches across studies.

Beyond substantial fitness benefits, especially concerning cardiorespiratory enhancements [e.g., (12, 15, 19, 20, 25, 26)], little is known about whether and how individuals experience these adaptations. In particular, scarce evidence exists about how exercise at different intensity domains (i.e., moderate and severe) and with different training prescriptions (i.e., HIIT and MICT) relates to subjective experiences of physical fitness, whether people actually feel fitter after exercising for a prolonged period, and what individual fitness experiences look like. In a previous study with the same sample, we were able to illustrate that physiological adaptations and subjective experiences of physical fitness correlate and that training modes (i.e., HIIT and MICT) and their different sequential order (i.e., first HIIT and then MICT or first MICT and then HIIT) may influence both physiological and subjective outcomes (6). While these results are in line with previous research suggesting that physiologically assessed and self-rated (i.e., subjectively perceived) physical fitness measures correlate (27, 28), they merely describe statistical associations without providing further explanations.

Therefore, qualitative analyses that assess subjective physical fitness experiences over the temporal course of a structured exercise intervention and with different training modes are warranted. Such analyses can “focus on subjective meanings and contexts” (29, p. 61) as they engage with individuals' experiences within particular settings. Concerning HIIT, only a few studies have qualitatively examined general experiences within an intervention setting (e.g., 30–32), and no studies have explicitly focused on the question of how physical fitness, as a particular outcome of regular exercise (33), is subjectively experienced over time. In our study, we focus on the experiences of physically inactive young adults. This group is of particular interest with regard to health promotion for at least two reasons. First, as Biddle has argued, public health promotion aims to help those who are least active to pursue a more active lifestyle (7). Second, healthy young adults find themselves in a period of their lives when PA and exercise behaviors often tend to decline or be abandoned due to critical life events and transitions (34) but may still be susceptible to individually tailored PA and exercise offers.

The purpose of this study is to explore how physically inactive young adults subjectively experience the development of physical fitness over time as they start to exercise. More specifically, we want to assess whether fitness experiences differ across different training modes (i.e., HIIT and MICT) and whether different sequences of these training modes (i.e., HIIT–MICT and MICT–HIIT) have an impact on how young adults perceive their physical fitness development. In this regard, the following questions guided our research process:

– How do inactive young adults subjectively experience the development of physical fitness over time when they start to exercise regularly?
– What role do training modes (i.e., HIIT and MICT) at different intensity domains (i.e., moderate and severe) play with regard to subjective experiences of physical fitness over time?
– What role does the sequence of different training modes (i.e., HIIT–MICT and MICT–HIIT) at different intensity domains (i.e., moderate and severe) play with regard to subjective experiences of physical fitness over time?

## 2. Materials and methods

### 2.1. Study design, setting, and protocol

This study is a result of a subproject of the transdisciplinary Individual Response to Physical Activity (iReAct) study, which comprised five study modules with researchers from the fields of sport sociology, exercise psychology, exercise physiology, biochemistry, and psychosomatics [for details, please see the study protocol (35)]. The primary goal of the iReAct project was to examine how physically inactive young adults (20–40 years) individually and biopsychosocially respond to an exercise intervention. The study was designed as a 15-week, two-period sequential exercise intervention comparing individual responses to two different endurance-based training modes (HIIT vs. MICT) and their sequential order (HIIT–MICT vs. MICT–HIIT). After one week of biopsychosocial baseline assessments (*t*_0_), participants were randomly assigned (based on VO_2max_ and sex) to either the HIIT or the MICT training group in which they subsequently trained for six weeks with three training sessions per week on a bicycle ergometer. The MICT group performed sixty minutes of continuous cycling at a power output corresponding to 90% of the first lactate threshold to make sure that participants were exercising at a moderate intensity (9, 10, 20). The HIIT group performed a ten minute warm-up at a power output corresponding to 70% of the maximal heart rate (HRmax), which was followed by 4 × 4 min intervals at a power output corresponding to 90% HRmax, which was above the second lactate threshold for all participants and therefore within the severe intensity domain (10, 20). Intervals were interspersed by 4 min resting periods at 30 Watts. HIIT sessions were concluded with a five minute cool-down period at 30 Watts. To complete the study protocol, participants needed to accumulate at least 15 training sessions across the six weeks. Training sessions took place at gyms at the Institute of Sports Science and the Department of Sports Medicine and were supervised by trained personnel. Participants' heart rates and electrocardiogram (ECG) were constantly monitored during the training sessions. Heart rate data was interpolated second-by-second for each training session and subsequently averaged into five second bins for an entire training week. Subsequently, the weekly average heart rate data was compared. If the mean heart rate in the following week was below a pre-defined threshold (here, −3 bpm), training intensity was increased by five Watts (for further details, please see (20)). After one week of biopsychosocial assessments (*t*_1_), participants switched the training modes and exercised for another six weeks. Afterwards, we conducted the final assessments (*t*_2_).

### 2.2. Participants

We aimed for a sample of healthy young adults who did not meet the WHO's current recommendations for regular PA [for a more detailed overview of the inclusion criteria, please see (35)]. After obtaining ethical approval from the “Ethics Committee of the Medical Faculty University Tübingen” (reference number: 882/2017BO1), participants were recruited using different communication channels, such as the university and the university clinics mailing lists, an experimental database, newspaper articles, and flyers. Before their enrolment, all participants were informed in detail about the study protocol and all associated risks and gave their written consent to participate. The study was conducted in six consecutive waves from 2018 to 2020. For this sub-study, a total of 31 (23 women and 8 men) participants with a mean age of 27 years (SD = 6; range = 20–40), of whom most were enrolled at the university, completed the entire study protocol (*n*_HIIT–MICT _= 16 and *n*_MICT–HIIT _= 15) (for more details on the inclusion process, please see (6)).

### 2.3. Data collection

The data relevant to this sub-study was collected at the end of the exercise intervention (*t*_2_) so that participants had exercised either in the sequence HIIT–MICT or MICT–HIIT and were able to comparatively evaluate their experiences on the effectiveness of both training modes. Strictly speaking, our participants can, therefore, not be described as inactive anymore. Instead, they started to exercise regularly after a prolonged period of inactivity and must therefore be considered to be *formerly* inactive.

To assess subjective experiences of physical fitness and its development over time, we conducted interviews using the biographical mapping method (see 36–38). This is a mixed-method approach which combines a semi-structured interview and a drawing activity in order to obtain qualitative data on experiences related to PA and exercise, physical fitness, and health, as well as quantified accounts of those experiences over time [see also (6), for an overview of all collected variables, please see Supplementary Table S1]. The biographical mapping method is firmly rooted in a relativist and constructivist paradigm, assuming that reality is multiple, malleable, subjective, and, therefore, dependent on those interpreting it. Therefore, this approach aims to retrace and better understand re-constructed subjective experiences relating to a particular research question (see also 39).

The interviews centered around the main feature of the biographical mapping method, which is a coordinate system that consists of a horizontal timeline (representing the intervention period in days over both training blocks) and a vertical intensity scale (ranging from zero to ten) and which is displayed for participants on a large touchscreen using the bioMAP-software (38, 40). To provide some temporal orientation, the interviewer already highlighted all training sessions of the two training periods beforehand. The procedure of the method is twofold and follows along a semi-structured interview guide, which leaves space for individual follow-up questions. Initially, participants were asked whether any relevant life events or daily hassles that impeded their daily routines occurred throughout the exercise intervention. The interviewer wrote down these events on the timeline and encouraged participants to talk about them and everything else they considered relevant or essential in the last weeks. Participants were also asked whether they had any particular expectations before enrolling in the study and to what extent these expectations were met or not. In a subsequent step, participants were asked to draw developmental curves for 14 activity- and health-related status and behavioral variables. In this context, the intensity scale mentioned above offered the possibility to express to which degree (i.e., 10 for very high and 0 for very low) those variables were situationally and contextually experienced at different times during the intervention. Two curves were explicitly related to physical fitness, one covering the perceived exercise-related physical fitness (i.e., training status) and the other covering the perceived physical fitness in everyday life (i.e., general fitness for everyday activities). While drawing, participants were encouraged to orient themselves towards the time scale, think aloud, comment on their experiences, and describe and explain the curve trajectories, slopes, and transitional periods.

HG conducted all interviews in an office at the Institute of Sports Science. Participants were already familiar with the procedure since the same method was used at baseline (*t*_0_) for a biographical life span analysis and before they switched training modes (*t*_1_) for a retrospective evaluation of the first training period. All participants gave their consent to audiotape the interviews. After the interviews, HG took field notes and filled out a protocol sheet for documentation purposes. The interviews ranged from 21 to 65 min (*M* = 41; SD = 12).

### 2.4. Data analysis

In order to identify *patterns* of how participants experience physical fitness and its development throughout a structured exercise intervention and the relationship to particular training intensities (i.e., moderate vs. severe) and modes (i.e., HIIT and MICT) and their different sequential order (i.e., HIIT–MICT and MICT–HIIT), we conducted a reflexive thematic analysis (41, 42). Our analysis focused solely on the verbal accounts and not on the visualizations of the curve trajectories. In the analytical process, we acknowledge researcher subjectivity as a resource and consider themes to be products of the decisions we have made along the analytic way (41, 43, 44).

HG and research assistants transcribed the interviews verbatim. The transcripts were then imported into MAXQDA (45). HG conducted the thematic analysis but regularly met with JJ and AT to discuss previous and subsequent analytic steps and preliminary codes, themes, and results. As a first step, HG familiarized himself with the data by reading the transcripts and taking memos and notes. After data familiarization, he coded the data set, which was an organic and recursive process (43) in that he went back and forth in the data set and (re-)adapted codes. Coding was done primarily inductively, meaning no codebook was used. In addition, HG aimed to code the data set on a semantic and a latent level. However, as coding progressed, most codes turned out to be rather descriptive. Referring back to the data set and the particular research questions and discussing with AT and JJ, we gained the impression that using the biographical mapping method over the shorter temporal period of an exercise intervention may have led participants to describe curve trajectories often on a superficial level. This stands in contrast to the life span-oriented perspective for which we usually employ the biographical mapping approach, where participants tended to reflect on their experiences in more detail, which might also be due to a greater number of critical life events and transitions. Therefore, we decided to recur to a qualitative descriptive methodological approach and aimed to give a comprehensive summary of experiences of physical fitness and its development over time by staying close to the data and providing straight descriptions of the phenomenon (cf. (46)). This was similarly done by Stork et al. (30), who aimed to assess young adults' experiences of HIIT, MICT, and sprint interval training (SIT). The intention to “stay closer to the data as given” (47, p. 78) does not mean that it is not interpretatively analyzed. In fact, Sandelowski (46) has pointed out early on that “all description entails interpretation” (p. 335). In this regard, while we decided to stay closer to the explications participants offered, we still aimed to interpretatively analyze their physical fitness experiences over time within a structured exercise intervention. After a second-round of coding and re-checking to counterbalance “coding drift’ (43), codes were assorted and clustered according to their meaning to develop initial candidate themes and subthemes, which were referred back to the research questions. In order to organize the candidate themes visually, a thematic map was developed. In a subsequent step, HG reviewed the potential themes and checked them against the codes and, subsequently, the data set. Similar to coding, this was recursive with the goal to develop themes that have a central organizing concept and are internally consistent. Eventually, theme definitions were written up and the themes were named (41, 43, 48).

### 2.5. Criteria for judging research quality

As our research is framed by ontological relativism (and thus acknowledges both participant and researcher subjectivity), a *criteriological approach* (49) to assess the quality or the *legitimacy* (50) of our data and analysis is deemed unfruitful and misleading. In contrast to a foundational view that uses a pre-determined and fixed set of criteria, we think of criteria as characteristics of our research that “can change depending upon the context and the purposes” (49) of a given study. Referring to Tracy (51) and Smith and Caddick (29), we decided on five criteria that align with our ontological position and study: worthy topic, substantive contribution, rich rigor, transparency, and coherence.

We consider our topic to be worthy, given that current public health initiatives seem to fail to sustainably motivate people to be more active, as indicated by an evidently low prevalence in global PA-levels (4, 5). A more profound knowledge of how individuals experience physical fitness as one of the most prominent physiological outcomes of regular exercise as well as its development might be helpful to understand whether young adults actually experience the supposed physiological benefits of training and how these experiences relate to different training modes and intensities. In this regard, our research may contribute to both the scientific community and public health practitioners as theoretical and practical implications can be derived. As further quality criteria, we attended to rich rigor, transparency, and coherence. Our sample and procedure were chosen to generate meaningful insights about a group that is particularly prone to decrease PA and exercise levels, namely young adults. The study preparation was thoroughly conducted, including several pilot runs for the biographical mapping interviews. In this regard, we further adjusted the curves and refined our interview guide. Throughout the data collection and analysis, HG documented his steps in the form of field protocols and images (e.g., for the thematic analysis) and kept a trail of the single analytic steps.

Additionally, we tried to achieve coherence by deriving our research questions from previous literature and current observations on the phenomenon of PA, exercise, and physical fitness and by aligning our methods with our research interests. In addition, from coding to theme development and refinement to drafting the final report, HG regularly met with JJ and AT, who served as “critical friends”, and presented his ideas. The aim of these interactions was not to reach a consensus on particular instances but to discuss possible interpretations, challenge each other's perceptions, receive feedback, and, thus, increase reflexivity (52).

## 3. Results and discussion

**Figure 1:**
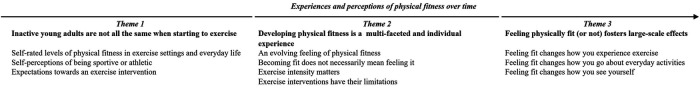
Overview of the themes describing experiences and perceptions of physical fitness over time.

As a result of our reflexive thematic analysis, we developed three overarching themes: (1) Inactive young adults are not all the same when starting to exercise; (2) Developing physical fitness is a multi-faceted and individual experience; and (3) Feeling physically fit (or not) fosters large-scale effects. These themes represent the temporal and processual character of starting to exercise after a prolonged period of inactivity, building up an exercise routine and potentially improving physical fitness, and experiencing the behavioral and experiential implications of feeling fit or not (see Figure 1). The following section presents these themes and related subthemes in detail. We refer to participants by their pseudonymized names, training sequences, and age.

### 3.1. Theme 1: Inactive young adults are not all the same when starting to exercise

This theme describes how inactive young adults who start to exercise within a structured exercise intervention setting differ in how they rate their initial levels of physical fitness concerning both exercise and everyday life activities, how they perceive themselves as sportive or athletic, and the expectations they have towards an exercise intervention with regard to physical fitness. In this regard, inactive young adults are not a homogenous group and hold disparate perceptions and expectations.

#### 3.1.1. Self-rated levels of physical fitness in exercise settings and everyday life

Most participants rated their exercise-related physical fitness levels relatively low at the beginning of the study and explained this mainly by an extended period of physical inactivity, which was expected and consistent with research that shows that a dose-response relationship between PA and fitness exists (53, 54). One participant stated, “Yes, so maybe I’m just so critical, but I felt my training condition was very bad at the beginning” (Gina, MICT–HIIT, 35), while another one explained, “So I would say that I didn’t do any sports for months before that or actually nothing at all for about a year” (George, MICT–HIIT, 26). In contrast, only a few participants rated their physical fitness levels as not low but mediocre.

On the other hand, physical fitness concerning everyday activities was generally perceived as higher. Most participants argued that they had no trouble mastering their everyday life and had no impairments considering physical functioning (e.g., walking or cycling for transportation, doing chores, or climbing the stairs). This finding is in line with the participant inclusion criteria of our study, as participants had to be healthy and capable of sustaining a physically strenuous exercise intervention for a prolonged period. Further, this finding aligns with previous research indicating that healthy adults generally score relatively high in questionnaires related to health (e.g., SF 36, WHOQOL-100, or WHOQOL-BREF) and associated subscales related to physical functioning (55–57). However, some participants also argued that although they felt physically capable, there was still room for improvement. For example, minor aches might impede feeling fit without any constraints, as one participant expressed, “So, I feel generally fit. So, as I said last time, I sometimes have back pain up here. But that's not (..) my performance is a little bit impaired” (Sabrina, HIIT–MICT, 26).

#### 3.1.2. Self-perceptions of being sportive or athletic

While previous physical inactivity was considered the main reason for low levels of exercise-related physical fitness, a few participants offered deeper explanations that related to their self-perception as active or inactive persons arguing that they were not athletes or jocks. Describing herself, one participant said, “I was at the beginning, so, I was very unathletic. I would say at a 3, simply because I was or am not necessarily the sport cannon, except for going shopping or so” (Laura, MICT–HIIT, 40). Similarly, another participant elaborated, “I would say (.) so. I’m not an athlete, but in everyday life it's alright” (Gillian, MICT–HIIT, 29). However, not seeing themselves as athletes or exercisers might be closely related to the explanation mentioned above of being insufficiently active. In this regard, research on exercise identity, for example, has shown that levels and pursued types of exercise are closely linked to the extent that individuals identify with exercise as an integral part of their self-concept (58, 59). Thus, considering oneself not sportive or athletic might subsequently lead to an avoidance of exercise-related behaviors and consequently to lower perceived physical fitness.

#### 3.1.3. Expectations towards an exercise intervention

Participants described two kinds of expectations they had concerning physical fitness. While a majority argued that they wanted to “become” fitter, more sportive, or trained, some stated that they wanted to “feel” this way instead. In hindsight, one participant reflected, “So my expectation was that I would *become* [emphasis added] more athletic” (Emma, MICT–HIIT, 23), while another participant argued, “Well, I expected to *feel* [emphasis added] a bit fitter” (George, MICT–HIIT, 26). These statements might reflect two different understandings of physical fitness. On the one hand, “being” or “becoming” fit might relate to a factual, physiological state that is measurable but may or may not be explicitly experienced (e.g., to be able to master an activity-related task). “Feeling” fit, on the other hand, might relate to a sensation that is actually perceived, for example, in the way that exercise sessions become easier or that participants can walk and cycle faster in their everyday life (see also Theme 3). However, participants might have also referred to more tangible outcomes, such as weight loss, which is often mentioned as an outcome expectation of exercise adoption (60).

In general, however, most participants stated that they had no expectations regarding physical fitness outcomes in particular but wanted to establish an exercise routine after prolonged physical inactivity. In this regard, a primary reason for participants to enroll in the study was not to become or feel fitter but to become physically more active within a structured setting that held them accountable to exercise consistently. One participated said, “So for me the main thing was that I get used to doing something regularly again” (Allison, HIIT–MICT, 23). Another participant went into more detail,

“That I have an incentive to overcome my weaker self, or rather that it is simply the case that I have a firm commitment and it doesn’t even come to the point, ‘Yes, but I could do something else’”. (Melanie, MICT–HIIT, 27)

These findings are in line with Larson et al. (60) who found that participants “expected that the formality of signing up for a research study would give them the accountability needed to exercise consistently for the full length of the programme” (p. 395).

### 3.2. Theme 2: Developing physical fitness is a multi-faceted and individual experience

This theme focuses on how inactive young adults do or do not experience improvements in their physical fitness when starting to exercise and how these experiences relate to particular training modes and their sequential order. The theme describes how a general feeling of physical fitness evolves across the entire intervention period independent of training mode, whether changes in physical fitness are palpable, what role exercise intensities play in this context, and the limitations a structured exercise intervention has.

#### 3.2.1. An evolving feeling of physical fitness

Most participants felt that their exercise-related physical fitness increased over the entire intervention period. However, some participants also indicated that they felt no or only minor changes in their fitness levels, which might be due to not achieving a personal goal, such as living up to a specific body ideal. One participant explained this non-development as follows,

“Continuously the same, not so good. (8s) Maybe because I also associate a certain body image with being trained, for example, where I say that I don’t have the feeling that I look particularly trained (..) And that's why I would say it's rather low”. (Sebastian, HIIT–MICT, 30)

A few participants also indicated that, in order to feel fit, they needed to exercise regularly. However, follow-up assessments and life events or other external factors disrupted the regularity of training. Although participants had to participate in an incremental and a reference test during follow-ups, this interruption of the everyday training routine was experienced as a period of inactivity, which led to decreased feelings of fitness. One participant noted,

“Because I have not trained, I have maybe somehow not felt so trained. So I would always associate this feeling of being trained with the fact that I’m also somehow in training right now, maybe”. (Jack, MICT–HIIT, 30)

In addition, becoming fit was not a linear experience but was impeded by disruptions, such as illness or pain, which made everyday activities particularly exhausting. This is consistent with research indicating that daily hassles and uplifts can perturbate behavioral patterns and how individuals feel (61, 62). However, these instances were usually momentary and did not impede the overall experience.

Physical fitness concerning everyday activities was usually rated as relatively high without much variation. Yet, some participants nonetheless experienced improvements in this regard.

“So, it was really better in between with being out of breath. So, I had said at the beginning, I’m going (unintelligible) up the stairs (both grin), yes and I easily feel 50 years older. That was really a little bit better then (…). But then I hadn’t noticed it so directly. I only noticed it again now, towards the end, where I thought, ‘Ah yes, that's going quite well now’. So, that has not changed now substantially between the two blocks I would say. So that actually is much better now”. (Sandra, HIIT–MICT, 37)

#### 3.2.2. Becoming fit does not necessarily mean feeling it

Participants did not always and immediately feel improvements in their physical fitness. Several participants described that they did not feel a training effect or that changes in their exercise-related physical fitness were not palpable. In these instances of uncertainty, “objective” feedback helped most participants grasp their individual progress. When they had no sense of whether they had improved their physical fitness, the physiological assessments at follow-ups I and II provided orientation and the affirmation that they were physiologically improving. In this regard, a participant stated,

“And then it was cool to see in the middle and during the diagnostics and now also on Monday during the incremental test that I have actually improved. And I had no idea how much I would somehow improve or something, so I am just happy that I have improved”. (Amber, MICT–HIIT, 22)

Another “objective” feedback marker was increased individual training load, which was similarly crucial to participants who could feel their progress. For them, individual load adjustments were an essential part of the training experience, as it confirmed “objectively” what they were experiencing “subjectively” and kept them motivated. One participant explained,

“So, you can then already somehow see every week or every second week, ‘Okay, I’m also making a little progress here. So again, okay, again 5 Watts more and then again 5’, so, yes, this then somehow rather motivates me”. (Jack, MICT–HIIT, 30)

In this regard, participants were sensitive towards training adjustments. This is noteworthy, as it might not be enough for participants to feel a difference; instead, they need to see one as well (60). In addition, these findings are consistent with research reporting that participants can experience (positive) progress monitoring as a crucial motivating factor to exercise adherence (31, 63). Once experienced, however, some participants also doubted their progress if there were no adjustments for a longer period or if adjustments occurred more often or more regularly in one training period than in the other. One participant reflected,

“And, otherwise, in general, I would say that I expected the continuous method, uh, that I expected something different. Because the intensity always increased during the interval training, I thought it would be the same with the continuous method”. (Joanne, HIIT–MICT, 25)

#### 3.2.3. Exercise intensity matters

Most participants experienced HIIT as more effective for their physical fitness than MICT. For them, the training effects of HIIT were more palpable, and they experienced greater increases in physical fitness in a shorter amount of time. On the other hand, MICT was mainly deemed ineffective and less beneficial, as some participants stated that they had felt no progress and that fitness improvements were not apparent despite a higher exercise volume. Comparing HIIT and MICT, one participant stated,

“I just had the feeling that the interval training is more beneficial. In other words, it was also more fun for me. The other one was boring. It was so long, like an hour, it dragged on. And, yes, somehow you didn’t see the progress in the other one, exactly”. (Kirsty, HIIT–MICT, 20)

These experiences are consistent with physiological research indicating that HIIT might foster greater enhancements in cardiorespiratory fitness than MICT (19, 25, 64). Several participants reasoned that the severe intensity of HIIT substantially contributed to a positive fitness experience as they associated exhaustion during and after the training session with a feeling of being activated and having accomplished something and considered it a positive sign for adaptation and improvement. One participant argued, “I think that also worked quite well. I think also because I found the interval training to be more strenuous and that's why you feel like you’ve done more afterwards” (Viola, MICT–HIIT, 20), while another one added, “that if you work harder, clearly you become a bit more trained” (Laura, MICT–HIIT, 40). This is consistent with findings suggesting that the ability to withstand severe intensities during HIIT provides participants with greater perceived benefits (30) and that a sense of achievement and satisfaction might be a result of mastering the difficulty and intensity of HIIT (31, 63). In contrast, MICT was not perceived as strenuous enough to always foster benefits related to fitness improvements. Most participants also experienced only minor to no fitness benefits as the moderate intensity of MICT was not difficult to manage and did not lead to exhaustion,

“Well, it's just that you don’t really get tired from the training, that is, from the continuous training, and then you’re actually like you’re going for a walk, so to speak. After a walk you also wouldn’t say, ‘oh, I’m trained now’ (laughs)”. (Mandy, HIIT–MICT, 27)

Most participants thus described MICT to be less effective and beneficial regarding their physical fitness, which they subsequently associated with less enjoyment and fun. However, a couple of participants also pointed out that they had experienced MICT to be effective nonetheless, which is in line with research suggesting that both HIIT and MICT can improve physical fitness (19). In particular, those who started with MICT felt improvements and stated that being active promotes fitness in any case. This might be because participants were previously inactive and had just started to exercise in this study. Exercising at a moderate intensity might have been perceived as effective as even small increases in regular moderate exercise can foster cardiorespiratory fitness benefits for previously insufficiently active people (65).

The tendency that HIIT was considered more effective than MICT was also reflected in participants' descriptions of their fitness development across the HIIT–MICT and MICT–HIIT sequences. Concerning exercise-related physical fitness, participants who started with HIIT described a substantial increase in the first training period, while the second training period was characterized by stable or even decreasing fitness levels. For the first training period (HIIT), one participant reflected, “But then it actually went quite uphill. So, especially the interval training felt good, so that you had the feeling, ‘I’m already fit somehow’” (Jessy, HIIT–MICT, 20), while other participants described the second training period (MICT) as follows, “And here I would simply draw a plateau, because (…) I don’t think that I have become significantly worse. But I haven’t noticed any major increase now either” (Ruby, HIIT–MICT, 21) and “so I had the feeling that this continuous method, that I even lose progress a bit. Even if I had a higher wattage afterwards” (Mandy, HIIT–MICT, 27). Only a few participants argued that physical fitness also increased in the second training period (MICT), although most of them also indicated that the rate of change was lower than during HIIT,

“Yes, I also feel a bit more trained. But I have to say, that it has increased rather less steeply, because you do the same thing over and over again and you have somehow, so I think, you feel at the higher intensity more that ‘Oh now, now it's going better’. And here then rather not so fast (I: So moderately increasing), because, yes, exactly”. (Anne, HIIT–MICT, 20)

On the other hand, if participants started with MICT, they also reported increases in exercise-related physical fitness more often for MICT, “That definitely went up during the first training block” (Viola, MICT–HIIT, 20). Additionally, while in the HIIT–MICT sequence, the second training period resembled more a plateau and sometimes even a regression in feeling fit, the second training period in the MICT–HIIT sequence was often perceived as relatively positive as participants experienced a continuous increase or even a boost in physical fitness. One participant stated, “And then at the high intensity, it took another big swing with my training condition” (Emma, MICT–HIIT, 23).

Eventually, several participants stated that their physical fitness had improved independently of the selected training modes. Those participants often described a rather linear increase in physical fitness across the entire intervention period, as indicated by the following statement,

“Ah, that's really hard to tell. I actually think that they are somehow even. Although I actually thought at first that the interval training might be more beneficial, because I thought it was somehow a bit more strenuous and so. But actually, from the sensation how trained I actually feel, it was not the case”. (Amber, MICT–HIIT, 22)

Regarding everyday physical fitness, participants did not elaborate much on the perceived effects of the two training modes. Only a few participants described a similar tendency as for the exercise-related physical fitness development, namely, increases during the first training period of the HIIT–MICT sequence, followed by a subsequent plateau or decrease in the second training period. Similarly, a few participants described increases in their everyday fitness during the first training period of the MICT–HIIT sequence, followed by an increase or even a boost in the HIIT period.

#### 3.2.4. Exercise interventions have their limitations

Some participants did not perceive the exercise intervention solely as positive. One of the main reasons was that exercising only on a bicycle ergometer was perceived to be too one-sided to improve fitness on a global level. While most participants reported increases in their physical fitness, they related these changes primarily to increased muscle mass in the upper thighs and improved cardiorespiratory fitness. However, to feel fitter on a more global scale, participants missed training other muscle groups (e.g., in the upper body),

“What bothered me a bit, because I come more from running, that it was concentrated only on the legs. So, the rest of the body felt so flabby (laughs) and the legs were really strong and pumped up”. (Carol, MICT–HIIT, 37)

This indicates that the experience of fitness and what it means to be fit or not is highly subjective and may expand on various concepts and notions of fitness, such as cardiorespiratory and muscular endurance, muscular strength, or body composition, which, however, might not be achieved by cycling alone. Although participants did not often speak explicitly about their body image, on a latent level, they still drew on socio-culturally determined body ideals, which were associated with a thin body for women and a muscular or athletic appearance for men (66, 67). This is in line with qualitative research indicating that subjective constructions of physical fitness often conflate with individual body images and how young people talk about themselves (68–70).

Additionally, participants argued that the intervention period was too short and the training volume was too low in order to feel trained on a more global level,

“I mean, as I said, I am (unintelligible) was and am not an athletic person and of course, somehow, in a few weeks you do not suddenly become a super athlete or at least not with 3 times a week of an adapted training”. (Ruby, HIIT–MICT, 21)

### 3.3. Theme 3: feeling physically fit (or not) fosters large-scale effects

This theme focuses on descriptions of the effects of feeling fit (or not), especially concerning its impact on exercise experiences, how participants go about their everyday activities, and how feeling fit changes how they see themselves.

#### 3.3.1. Feeling fit changes how you experience exercise

Feeling that exercise-related physical fitness improved positively affected how participants experienced the exercise sessions. While HIIT was initially described as particularly strenuous and exhausting, several participants indicated that physical adaptations made the training sessions easier (even though the external load was adjusted over time). One participant elaborated, “So at the beginning it was just, I would say very strenuous and then, of course, you also saw successes, that's why this increase at the beginning. Then it became better of course” (Sandra, HIIT–MICT, 37). In particular, participants stated that they “suffered” less, were less exhausted during the high-intensity intervals, could withstand exhaustion for a longer time, and recovered faster in between intervals. A participant explained her experience as follows,

“I have actually felt more trained there. With time, because you also notice how the intervals with the higher frequency, how you then become better and less exhausted”. (Anne, HIIT–MICT, 20)

This is in line with previous findings that suggest that despite lower training volume, for example, compared to MICT, HIIT leads to substantial improvements in exercise tolerance over time (14) and might become more habitual (63) as individuals begin to feel fitter and can sustain the high intensities of HIIT for a longer period (71). Several participants also explained that they increasingly enjoyed HIIT as physical fitness adaptations were palpable. One stated,

“But, yes, then it was also more fun and you also noticed that you recover faster in the recovery phases or so. So, the intervals were still strenuous, I thought, but the recovery was faster and then it was also more fun”. (Oliver, HIIT–MICT, 37)

Consequently, a couple of participants stated that the training became more of a priority for them and that they were more motivated to train when they either felt that their fitness was improving or when the training load was increased. On an affective level, these results align with research reporting that, despite experiencing less pleasure in-task, HIIT can be equally or even more enjoyable than MICT, especially with regard to post-exercise enjoyment (72, 73). In addition, these findings are in line with research indicating that enjoyment for HIIT increases throughout an intervention period, while enjoyment for MICT remains relatively constant and lower (74). In this regard, Kinnafick et al. (31) have pointed out that mastering vigorous intensities can increase perceptions of competence and, thus, general positive affect. This might also be a reason why experiencing increases in physical fitness during the HIIT period led participants to be more motivated for the training. Kwan and Bryan (75) have similarly pointed out that improvements in affective responses are associated with exercise self-efficacy, which in turn might improve exercise motivation over time. Referring to Self-Determination Theory, Teixeira et al. (76) have pointed out that feeling autonomous and effective can promote intrinsic motivation and thus exercise adherence.

A few participants described similar adaptations to MICT, which was generally perceived as not as strenuous or exhausting and might have led to less joy and pleasure during the training session as there was no apparent progress. One participant explained, “With the continuous method, it's basically the same every time. And you do not feel a great training effect” (Silas, MICT–HIIT, 20).

#### 3.3.2. Feeling fit changes how you go about everyday activities

Although participants generally rated their levels of physical fitness related to everyday activities as high without much variation, some still experienced changes in this regard. Several participants reported that they were able to walk faster and had improved their “cycling ability”, for example, by being able to go in a higher gear or feeling less exhausted,

“So, I also notice that in everyday life, so for example I walked here again this morning and I thought, ‘oh dear, oh dear, I’m late’, because during the first week of diagnostics it took me about three quarters of an hour. And now it didn’t take me three quarters of an hour. I think it took me maybe half an hour to go the same distance”. (Emma, MICT–HIIT, 23)

Additionally, participants were not out of breath so quickly and recovered faster after PA.

Feeling fit and being able to master demanding everyday activities more easily also led to more PA in those participants, who felt that their fitness levels increased in this regard. Especially those few who had children reported that they had more energy in their everyday life and had become more active with their kids. One participant gave a vivid example,

“So, I’m imagining it. I don’t know if it's true, sure. Um, that it has already gotten better. I would increase that from 5 to 8 (…). Because the other day, I don’t remember when it was, I went out with my children. They really wanted to go out. And then we went 4.2, so we did a lap and I measured it. It was 4.2 kilometers, and after he half, they said they didn’t feel like it anymore. I was like ‘yeah, that was the first time they said they’re not up for it anymore (laughs)’ (I: Walking right?) and I could still. Yes, I walked and they just rode bikes and skateboards and I walked alongside. And climbing stairs. It's getting better, so I’m still out of breath, but it's getting better. I have the feeling not that I’m getting faster, but my breath is getting better”. (Laura, MICT–HIIT, 40)

#### 3.3.3. Feeling fit changes how you see yourself

Last but not least, feeling fit changed the way several participants saw themselves. Regular training was linked to changes in the body and appearance. The fitter participants felt, and by knowing that they were “doing something for their body”, the better and more comfortable they felt in their bodies. One participant explained, “And then, however, over the course of the training it already rose a bit. Because simply, so from the feeling, by exercising more and regularly I just felt better in my body” (Emma, MICT–HIIT, 23). In particular, a couple of participants reported increased muscle mass, which was associated with feeling powerful and capable. This is in line with research that has shown that PA and exercise interventions can positively impact individuals’ body image and self-perception (77). However, feeling more powerful and capable represents a less tangible and verifiable effect than, for example, losing weight (60). Additionally, one participant also reported that she rediscovered her self-confidence and a sense of self-efficacy, “I am again aware of what I can also achieve physically. (unintelligible) this lack of physical strength, but now I perceive it, so that it is there, I perceive it again” (Gina, MICT–HIIT, 35).

## 4. Strengths, limitations, and implications for future research and practice

### 4.1. Strengths and limitations of the study

To our knowledge, this is the first study to assess the processual character of fitness development in a structured exercise intervention from a subjective perspective. The methodological approach of our study can be considered a strength on several levels. First, the retrospective assessment of experiences across the intervention period using a biographical mapping method yields deeper insights, provides a contextualized understanding of exercise- and fitness-related experiences, and considers physical fitness and its development over time. Second, our research extends previous studies in that we did not solely focus on a comparison of HIIT and MICT but also compared the administration of two different sequential orders in which HIIT and MICT were prescribed (i.e., HIIT–MICT and MICT–HIIT), thus allowing participants to comparatively evaluate both training modes. Third, our sample presents a group that might be of particular interest to public health and PA policies since young and healthy inactive adults are a potential target group that might potentially be easy to reach as physical limitations might not yet impair the uptake of PA and exercise.

Our study also has some limitations. First, as indicated by the participants, our study design was limited in that the selected training modes primarily emphasized cardiorespiratory fitness development. Strength training was not included in the exercise prescription, which therefore did not meet current public health PA guidelines which usually include muscle-strengthening activities on a least two days a week (78). Comparing HIIT and MICT and their respective sequences and conducting the training solely on cycle ergometers might not be the training mode young and healthy adults might have chosen if they wanted to start exercising after a prolonged period of inactivity. Instead, as also indicated in the interviews, young people might start running or doing body-weight or resistance training (either in the gym or home-based) instead of sitting on a bicycle for forty to sixty minutes. Second, our findings might resonate particularly with inactive yet healthy young adults, who, in addition, were motivated to participate in an exercise study and were mostly female and students. In addition, our study setting was highly professional, with individual monitoring and regular check-ups of each participant. We are aware that exercise—in the real world—is not an option that is similarly available for all and that exercise is or cannot be a desirable or manageable behavior for everyone (79, 80). Similarly, feedback on individual progress, as provided in our study, is specific to an exercise intervention and might not be available outside of such contexts for those who cannot afford self-tracking devices or personal trainers. In this regard, our findings must be considered in our study's particular setting and with regard to the particular sample we assessed. Third, our interview guide for the biographical mapping interview was tightly designed as participants had several assessments on the same day and needed to change venues for clinical testing. Therefore, and since the biographical mapping covered a broad range of exercise- and health-related dimensions, a deeper analysis, for example linking individual experiences to social or structural issues, was not possible as participants reflected primarily on the experiences specific to the exercise intervention. Fourth, we did not conduct any follow-up assessments beyond the intervention period. Thus, we cannot make any statements as to whether our study helped participants be more active on their own. Finally, while we initially intended to recruit 60 participants for the iReAct study (35), nine participants dropped out because they were not able to complete the study protocol due to the imposed lockdowns in the wake of the COVID-19 pandemic. In addition, further data collection waves could not be realized due to the pandemic and associated restrictions.

### 4.2. Methodological implications and future research

Physical fitness is a complex and subjectively experienced phenomenon. Our results illustrate that participants perceive and experience physical fitness and its development in various ways. This inter-individual variance relates to what individuals associate with physical fitness; if, when, and how they feel their physical fitness improving; and how physical fitness affects their behavior and experience beyond the exercise setting itself. In a recent study on affective responses, Stork et al. (30) have concluded that inactive young adults “respond differently to different forms of exercise and the factors that influence participation in interval or continuous exercise are far more complex than can be captured by quantitative methodologies alone” (p. 10). Our results are in line with this conclusion. While in a previous quantitative study, we could identify some overlap between the improvements in physiological and subjectively experienced fitness (6), this qualitative analysis offered some possible explanations for those developments. Future research might further pursue qualitative approaches to better understand complex phenomena such as PA and exercise and associated (physical fitness) outcomes from a subjective viewpoint.

Participants from our study mostly favored HIIT over MICT and considered it more effective. Participants notably attributed fitness improvements to the severe intensity of HIIT and reasoned that a feeling of exhaustion during or after exercise was a sign of having accomplished something. In this respect, higher intensities may have elicited a sense of achievement, while post-exercise exhaustion may have been interpreted as a sign of having actively been engaged in exercise. However, it is essential to point out that exercise intensities need to be physiologically and psychologically manageable for participants, as studies have indicated that intensities beyond a critical threshold might lead to decreases in pleasure and, subsequently, in potential exercise adherence (81). In addition, some participants favored MICT over HIIT and considered it more effective. In this regard, Stork et al. (30) have proposed that “it would be more meaningful for future research questions to be framed in terms of ‘HIIT and/or MICT’” (p. 10) and avoid a HIIT vs. MICT ductus. This might strengthen HIIT as a viable alternative for individualized exercise prescriptions, but at the same time not dismiss MICT as an ineffective option *per se*.

Improving physical fitness and feeling those improvements has wide-ranging benefits related to exercise experiences and beyond. In particular, feeling fitter can change how young adults experience single training sessions. For example, physical adaptations led participants to enjoy HIIT more over time and to be more motivated for the exercise sessions. We think that palpable fitness adaptations might precede changes in affective responses to exercise. If young adults can actually feel the progress, they are making and thus experience some sense of mastery or self-efficacy, this can lead to more enjoyment and motivation. On the other hand, a lack of perceived progress might lead to less enjoyment and motivation (or even decreases) due to the experience of stagnation or monotony. To our knowledge, the iReAct-study is the first to assess and compare the prescriptions of different sequential orders of HIIT and MICT. Assessing different exercise modes in different sequential order might provide another avenue for future studies with regard to both physiological and experiential adaptations and their interrelationship.

Finally, as we have pointed out, no follow-up assessments took place to assess whether participants continued to exercise and which role their experiences with HIIT and MICT might have played in this regard. Of course, for meaningful improvements in physical fitness to occur, a continued adherence to PA and exercise is required. Future research might therefore further focus on long-term behavioral patterns of PA and exercise after an intervention period has been concluded. Herewith, repeated surveys over an extended time might be especially helpful to better understand the impact an intervention might actually have on individuals' PA and exercise behaviors.

### 4.3. Practical implications

Our study has implications for those administering exercise interventions in the context of both research as well as health and PA promotion. First, while there is a general tendency that young adults experience HIIT to be more effective and beneficial than MICT regarding physical fitness, not all participants prefer HIIT to MICT. Practitioners should acknowledge this and—similar to researchers—frame HIIT and MICT as alternative options in a pool of exercise opportunities rather than framing them as one vs. the other or merely highlighting HIIT as an extraordinarily potent training mode for everyone (82, 83). Similarly, it is important to note that the sequence of training modes matters when more than one method is administered. For practitioners, who might be more flexible in what exercise modes they administer (since they might not have to stick to a rigid study protocol), this might mean trying to “provide people with opportunities to engage in and try different forms of continuous and interval exercise” (72, p. 2120) to find an individually fitting exercise program.

Second, researchers and practitioners must be aware that physical adaptations are not similarly palpable to all participants. In this regard, providing feedback regarding physiological progress is crucial. Adjustments in the training load require constant monitoring of individual development over time but can reaffirm to participants that they are actually making progress, promote positive emotions, keep them motivated, and, thus, increase exercise adherence. However, if participants do not improve on a physiological level and training loads cannot be adjusted, exercise professionals should equally provide feedback and offer training alternatives since participants are well aware if no adjustments occur, which might limit perceived self-efficacy and motivation. Additionally, it appears relevant to promote awareness of subjective outcomes associated with increased physical fitness, such as feeling more powerful, capable, or energized for participants to *feel* the distinctive benefits of regular exercise.

Third, it is vital for researchers and practitioners alike to be aware that structured exercise interventions have their limitations. Our results have shown that participants have a complex understanding of the concept of physical fitness, which needs to be addressed if exercise interventions should be sustainable. For participants who aim to improve their overall physical fitness and well-being, exercise interventions focusing primarily on cardiorespiratory fitness and only selected muscle groups (e.g., cycling) might be less beneficial than other modes of exercise (e.g., body weight exercises or resistance training). This requires understanding physical fitness not merely as a physiological outcome but as a subjective construction.

## 5. Conclusion

In recent years, the discussion around PA and exercise promotion for public health purposes has focused, among others things, on the question of whether HIIT might be a viable option. Compelling evidence has shown that HIIT is associated with substantial increases in physical fitness. However, little is known about whether and how individuals actually experience these adaptations when they start to exercise. The objective of this study was to qualitatively assess how (inactive) healthy young adults subjectively experience physical fitness and its development throughout a structured exercise intervention in which they were prescribed HIIT and MICT in a different sequential order and how they retrospectively evaluate those experiences. Our results emphasize that physical fitness is a highly individualized and complex experience that manifests in various ways. Although participants generally seem to favor HIIT to be more effective than MICT, the idiosyncrasy of physical fitness experiences also reiterates that one-size-fits-all approaches might be misleading and that individual expectations, perceptions, and experiences need to be taken into account when administering training modes. We think that our findings can extend the knowledge about how young adults subjectively experience exercise and associated fitness outcomes and the role HIIT and MICT can play in this regard, thus strengthening vigorous exercise as a viable alternative to or an extension of moderate exercise programs, particularly in previously inactive young and healthy adults.

## Data Availability

The datasets presented in this article are not readily available because due to ethical/privacy reasons. General information on the data is provided by the authors upon reasonable request. Requests to access the datasets should be directed to Hannes Gropper, hannes.gropper@uni-tuebingen.de.

## References

[1] Warburton DER Bredin SSD. Health benefits of physical activity: a systematic review of current systematic reviews. Curr Opin Cardiol. (2017) 32(5):541–56. 10.1097/HCO.0000000000000437 28708630

[2] Rhodes RE Janssen I Bredin SSD Warburton DER Bauman A. Physical activity: health impact, prevalence, correlates and interventions. Psychol Health. (2017) 32(8):942–75. 10.1080/08870446.2017.1325486 28554222

[3] Morgan AJ Parker AG Alvarez-Jimenez M Jorm AF. Exercise and mental health: an exercise and sports science Australia commissioned review. J Exerc Physiol. (2013) 16(4):64–73.

[4] Guthold R Stevens GA Riley LM Bull FC. Worldwide trends in insufficient physical activity from 2011 to 2016: a pooled analysis of 358 population-based survey with 1.9 million participants. Lancet Glob Health. (2018) 6:e1077–e86. 10.1016/S2214-109X(18)30357-7 30193830

[5] Hallal PC BAL Bull FC Guthold R Haskell W Ekelund U. Global physical activity levels: surveillance progress, pitfalls, and prospects. Lancet. (2012) 380:247–57. 10.1016/S0140-6736(12)60646-1 22818937

[6] Gropper H Mattioni Maturana F Nieß AM Thiel A. Does becoming fit mean feeling (f)it? A comparison of physiological and experiential fitness data from the iReAct study. Front Sports Act Living. (2021) 3(244). 10.3389/fspor.2021.729090 34541523 PMC8440924

[7] Biddle SJH Batterham AM. High-intensity interval exercise training for public health: a big HIT or shall we HIT it on the head? Int J Behav Nutr Phys Act. (2015) 12(1):95. 10.1186/s12966-015-0254-9 26187579 PMC4506613

[8] Keating SE Johnson NA Mielke GI Coombes JS. A systematic review and meta-analysis of interval training versus moderate-intensity continuous training on body adiposity. Obes Rev. (2017) 18(8):943–64. 10.1111/obr.12536 28513103

[9] Poole DC Jones AM. Oxygen uptake kinetics. Compr Physiol. (2012) 2(2):933–96. 10.1002/cphy.c100072 23798293

[10] Keir DA Iannetta D Mattioni Maturana F Kowalchuk JM Murias JM. Identification of non-invasive exercise thresholds: methods, strategies, and an online app. Sports Med. (2022) 52(2):237–55. 10.1007/s40279-021-01581-z 34694596

[11] Hofmann P Tschakert G. Intensity- and duration-based options to regulate endurance training. Front Physiol. (2017) 8:337. 10.3389/fphys.2017.00337 28596738 PMC5442222

[12] Gibala MJ Little JP MacDonald MJ Hawley JA. Physiological adaptations to low-volume, high-intensity interval training in health and disease. J Physiol (Lond). (2012) 590(5):1077–84. 10.1113/jphysiol.2011.224725 22289907 PMC3381816

[13] Gillen JB Gibala MJ. Is high-intensity interval training a time-efficient exercise strategy to improve health and fitness? Interval Training. (2018) 01(01):409–12. 10.1139/apnm-2013-0187 24552392

[14] Gibala MJ. High-intensity interval training: a time-efficient strategy for health promotion? Curr Sports Med Rep. (2007) 6(4):211–3. 10.1097/01.CSMR.0000306472.95337.e9 17617995

[15] Martland R Mondelli V Gaughran F Stubbs B. Can high-intensity interval training improve physical and mental health outcomes? A meta-review of 33 systematic reviews across the lifespan. J Sports Sci. (2020) 38(4):430–69. 10.1080/02640414.2019.1706829 31889469

[16] Jung ME Locke SR Bourne JE Beauchamp MR Lee T Singer J Cardiorespiratory fitness and accelerometer-determined physical activity following one year of free-living high-intensity interval training and moderate-intensity continuous training: a randomized trial. Int J Behav Nutr Phys Act. (2020) 17(1):25. 10.1186/s12966-020-00933-8 32102667 PMC7045584

[17] Hu M Kong Z Sun S Zou L Shi Q Chow BC Interval training causes the same exercise enjoyment as moderate-intensity training to improve cardiorespiratory fitness and body composition in young Chinese women with elevated BMI. J Sports Sci. (2021) 39(15):1677–86. 10.1080/02640414.2021.1892946 33634738

[18] Taylor JD Fletcher JP Mathis RA Cade WT. Effects of moderate- versus high-intensity exercise training on physical fitness and physical function in people with type 2 diabetes: a randomized clinical trial. Phys Ther. (2014) 94(12):1720–30. 10.2522/ptj.20140097 25082918

[19] Maturana FM Martus P Zipfel S Nieß AM. Effectiveness of HIIE versus MICT in improving cardiometabolic risk factors in health and disease: a meta-analysis. Med Sci Sports Exerc. (2021) 53(3):559–73. 10.1249/mss.0000000000002506 32890201

[20] Maturana FM Schellhorn P Erz G Burgstahler C Widmann M Munz B Individual cardiovascular responsiveness to work-matched exercise within the moderate- and severe-intensity domains. Eur J Appl Physiol. (2021) 121(7):2039–59. 10.1007/s00421-021-04676-7 33811557 PMC8192395

[21] Mendelson M Chacaroun S Baillieul S Doutreleau S Guinot M Wuyam B Effects of high intensity interval training on sustained reduction in cardiometabolic risk associated with overweight/obesity: a randomized trial. J Exerc Sci Fit. (2022) 20(2):172–81. 10.1016/j.jesf.2022.03.001 35401768 PMC8956941

[22] Vaccari F Passaro A D’Amuri A Sanz JM Di Vece F Capatti E Effects of 3-month high-intensity interval training vs. moderate endurance training and 4-month follow-up on fat metabolism, cardiorespiratory function and mitochondrial respiration in obese adults. Eur J Appl Physiol. (2020) 120(8):1787–803. 10.1007/s00421-020-04409-2 32514607

[23] Vella CA Taylor K Drummer D. High-intensity interval and moderate-intensity continuous training elicit similar enjoyment and adherence levels in overweight and obese adults. Eur J Sport Sci. (2017) 17(9):1203–11. 10.1080/17461391.2017.1359679 28792851 PMC6104631

[24] Soylu Y Arslan E Sogut M Kilit B Clemente F. Effects of self-paced high-intensity interval training and moderate-intensity continuous training on the physical performance and psychophysiological responses in recreationally active young adults. Biol Sport. (2021) 38(4):555–62. 10.5114/biolsport.2021.100359 34937964 PMC8670818

[25] Milanović Z Sporiš G Weston M. Effectiveness of high-intensity interval training (HIT) and continuous endurance training for VO2max improvements: a systematic review and meta-analysis of controlled trials. Sports Med. (2015) 45(10):1469–81. 10.1007/s40279-015-0365-0 26243014

[26] Batacan RB Duncan MJ Dalbo VJ Tucker PS Fenning AS. Effects of high-intensity interval training on cardiometabolic health: a systematic review and meta-analysis of intervention studies. Br J Sports Med. (2017) 51(6):494–503. 10.1136/bjsports-2015-095841 27797726

[27] Monroe CM Thomas DQ Lagally K Cox A. Relation of college students’ self-perceived and measured health-related physical fitness. Percept Mot Skills. (2010) 111(1):229–39. 10.2466/06.07.13.Pms.111.4.229-239 21058602

[28] Hausenblas H Downs D. Comparison of body image between athletes and nonathletes: a meta-analytic review. J Appl Sport Psychol. (2001) 13:323–29. 10.1080/104132001753144437

[29] Smith B Caddick N. Qualitative methods in sport: a concise overview for guiding social scientific sport research. Asia Pacific J Sport Soc Sci. (2012) 1(1):60–73. 10.1080/21640599.2012.701373

[30] Stork MJ Williams TL Martin Ginis KA. Unpacking the debate: a qualitative investigation of first-time experiences with interval exercise. Psychol Sport Exerc. (2020) 51:101788. 10.1016/j.psychsport.2020.101788

[31] Kinnafick F-E Thøgersen-Ntoumani C Shepherd SO Wilson OJ Wagenmakers AJM Shaw CS. In it together: a qualitative evaluation of participant experiences of a 10-week, group-based, workplace HIIT program for insufficiently active adults. J Sport Exerc Psychol. (2018) 40(1):10–9. 10.1123/jsep.2017-0306 29521569

[32] Burn N Niven A. Why do they do (h)it? Using self-determination theory to understand why people start and continue to do high-intensity interval training group exercise classes. Int J Sport Exerc Psychol. (2019) 17(5):537–51. 10.1080/1612197X.2017.1421682

[33] Lin X Zhang X Guo J Roberts CK McKenzie S Wu WC Effects of exercise training on cardiorespiratory fitness and biomarkers of cardiometabolic health: a systematic review and meta-analysis of randomized controlled trials. J Am Heart Assoc. (2015) 4(7):e002014. 10.1161/JAHA.115.002014 26116691 PMC4608087

[34] Gropper H John JM Sudeck G Thiel A. The impact of life events and transitions on physical activity: a scoping review. PLoS One. (2020) 15(6). 10.1371/journal.pone.0234794 32569282 PMC7307727

[35] Thiel A Sudeck G Gropper H Mattioni Maturana F Schubert T Srismith D The iReAct study: a biopsychosocial analysis of the individual response to physical activity. Contemp Clin Trials Commun. (2020) 17. 10.1016/j.conctc.2019.100508 31890988 PMC6928277

[36] Schubring A Mayer J Thiel A. Drawing careers: the value of a biographical mapping method in qualitative halth research. Int J Qual Methods. (2019) 18:1–12. 10.1177/1609406918809303

[37] Thiel A John JM Carl J Thedinga HK. Weight stigma experiences and physical (in)activity: a biographical analysis. Obes Facts. (2020) 13(3):386–402. 10.1159/000507936 32604098 PMC7445546

[38] Thiel A Gropper H John JM Keppler V Mayer J. bioMAP: development of a software for the retrospective analysis of biopsychosocial health trajectories in elite sport. (2020). 10.15496/publikation-38957

[39] Guba EG Lincoln YS. Competing paradigms in qualitative research. In: Denzin NK Lincoln YS, editors. Handbook of qualitative research. 2. Thousand Oaks: Sage Publications (1994). p. 105–17.

[40] Thiel A Gropper H John JM Keppler V Mayer J. Development of a biographical mapping software (bioMAP) for the analysis of biopsychosocial health trajectories using the example of young top athletes: A brief report. Tübingen: University Library Tübingen (2020).

[41] Braun V Clarke V. Reflecting on reflexive thematic analysis. Qual Res Sport Exerc Health. (2019) 11(4):589–97. 10.1080/2159676X.2019.1628806

[42] Braun V Clarke V. Can I use TA? Should I use TA? Should I not use TA? Comparing reflexive thematic analysis and other pattern-based qualitative analytic approaches. Couns PsychiotherRes. (2020) 21(1):37–47. 10.1002/capr.12360

[43] Braun V Clarke V. Thematic analysis. In: Lyons E Coyle A, editors. Analysing qualitative data in psychology. 2nd edn. London: Sage (2016). p. 84–103.

[44] Braun V Clarke V. One size fits all? What counts as quality practice in (reflexive) thematic analysis? Qual Res Psychol. (2020) 18(3):1–25. 10.1080/14780887.2020.1769238

[45] Rädiker S Kuckartz U. Analyse qualitativer daten mit MAXQDA: text, audio und video. Wiesbaden: Springer VS (2019).

[46] Sandelowski M. Whatever happened to qualitative description? Res Nurs Health. (2000) 23(4):334–40. 10.1002/1098-240X(200008)23:4-334::AID-NUR9>3.0.CO;2-G 10940958

[47] Sandelowski M. What's in a name? Qualitative description revisited. Res Nurs Health. (2010) 33(1):77–84. 10.1002/nur.20362 20014004

[48] Terry G. Doing thematic analysis. In: Lyons E Coyle A, editors. Analaysing qualitative data in psychology. 2nd edn. London: Sage (2016). p. 104–18.

[49] Sparkes AC Smith B. Judging the quality of qualitative inquiry: criteriology and relativism in action. Psychol Sport Exerc. (2009) 10(5):491–7. 10.1016/j.psychsport.2009.02.006

[50] Burke S. Rethinking ‘validity’ and ‘trustworthiness’ in qualitative inquiry. In: Smith B Sparkes AC, editors. Routledge handbook of qualitative research in sport and exercise. London: Routledge (2019). p. 330–9.

[51] Tracy SJ. Qualitative quality: eight “big-tent” criteria for excellent qualitative research. Qual Inq. (2010) 16(10):837–51. 10.1177/1077800410383121

[52] Smith B McGannon KR. Developing rigor in qualitative research: problems and opportunities within sport and exercise psychology. Int Rev Sport Exerc Psychol. (2018) 11(1):101–21. 10.1080/1750984X.2017.1317357

[53] Oja P. Dose response between total volume of physical activity and health and fitness. Med Sci Sports Exerc. (2001) 33(6):S428–S37. 10.1097/00005768-200106001-00011 11427767

[54] Lee I-M. Dose-response relation between physical activity and fitness: even a little is good; more is better. JAMA. (2007) 297(19):2137–9. 10.1001/jama.297.19.2137 17507351

[55] Horner-Johnson W Krahn GL Suzuki R Peterson JJ Roid G Hall T. Differential performance of SF-36 items in healthy adults with and without functional limitations. Arch Phys Med Rehabil. (2010) 91(4):570–5. 10.1016/j.apmr.2009.12.015 20382289

[56] Perneger TV Leplège A Etter J-F Rougemont A. Validation of a French-language version of the MOS 36-item short form health survey (SF-36) in young healthy adults. J Clin Epidemiol. (1995) 48(8):1051–60. 10.1016/0895-4356(94)00227-H 7775992

[57] Bonomi AE Patrick DL Bushnell DM Martin M. Validation of the United States’ version of the world health organization quality of life (WHOQOL) instrument. J Clin Epidemiol. (2000) 53(1):1–12. 10.1016/S0895-4356(99)00123-7 10693897

[58] Anderson DF Cychosz CM. Development of an exercise identity scale. Percept Mot Skills. (1994) 78(3):747–51. 10.1177/003151259407800313 8084685

[59] Anderson DF Cychosz CM. Exploration of the relationship between exercise behavior and exercise identity. J Sport Behav. (1995) 18(3):159–66.

[60] Larson HK McFadden K McHugh T-LF Berry TR Rodgers WM. You can’t always get what you want: expectations, outcomes, and adherence of new exercisers. Qual Res Sport Exerc Health. (2017) 9(3):389–402. 10.1080/2159676X.2017.1294103

[61] Uijtdewilligen L Singh AS Chinapaw MJ Koppes LLJ van Mechelen W Twisk JWR. Number and appraisal of daily hassles and life events in young adulthood: the association with physical activity and screen time: a longitudinal cohort study. BMC Public Health. (2014) 14(1):1067. 10.1186/1471-2458-14-1067 25308800 PMC4213509

[62] Kanner AD Coyne JC Schaefer C Lazarus RS. Comparison of two modes of stress measurement: daily hassles and uplifts versus major life events. J Behav Med. (1981) 4(1):1–39. 10.1007/BF00844845 7288876

[63] Metcalfe RS Atef H Mackintosh K McNarry M Ryde G Hill DM Time-efficient and computer-guided sprint interval exercise training for improving health in the workplace: a randomised mixed-methods feasibility study in office-based employees. BMC Public Health. (2020) 20(1):313. 10.1186/s12889-020-8444-z 32164631 PMC7068982

[64] Sultana RN Sabag A Keating SE Johnson NA. The effect of low-volume high-intensity interval training on body composition and cardiorespiratory fitness: a systematic review and meta-aanalysis. Sports Med. (2019) 49(11):1687–721. 10.1007/s40279-019-01167-w 31401727

[65] Pavlik G Bakács E Csajági E Bakács T Noe J Kirschner R. Improved cardiorespiratory fitness following moderate exercise may encourage inactive people for doable and sustainable behavioral change. J Sports Med Phys Fitness. (2018) 59(3):502–9. 10.23736/s0022-4707.18.08043-x 29589406

[66] Fatt SJ Fardouly J Rapee RM. #Malefitspo: links between viewing fitspiration posts, muscular-ideal internalisation, appearance comparisons, body satisfaction, and exercise motivation in men. New Media Soc. (2019) 21(6):1311–25. 10.1177/1461444818821064

[67] Tiggemann M Zaccardo M. ‘Strong is the new skinny’: a content analysis of #fitspiration images on Instagram. J Health Psychol. (2018) 23(8):1003–11. 10.1177/1359105316639436 27611630

[68] Wright J O’Flynn G Macdonald D. Being fit and looking healthy: young women’s and men’s constructions of health and fitness. Sex Roles. (2006) 54(9):707–16. 10.1007/s11199-006-9036-9

[69] Harris J Cale L Duncombe R Musson H. Young people’s knowledge and understanding of health, fitness and physical activity: issues, divides and dilemmas. Sport Educ Soc. (2018) 23(5):407–20. 10.1080/13573322.2016.1228047

[70] Powell D Fitzpatrick K. ‘Getting fit basically just means, like, nonfat’: children's lessons in fitness and fatness. Sport Educ Soc. (2015) 20(4):463–84. 10.1080/13573322.2013.777661

[71] Burn NL Weston M Atkinson G Graham M Weston KL. Brief exercise at work (BE@work): a mixed-methods pilot trial of a workplace high-intensity interval training intervention. Front Sports Act Living. (2021) 3:699608. 10.3389/fspor.2021.699608 34278300 PMC8282817

[72] Stork MJ Gibala MJ Martin Ginis KA. Psychological and behavioral responses to interval and continuous exercise. Med Sci Sport Exer. (2018) 50(10):2110–21. 10.1249/mss.0000000000001671 29771824

[73] Stork MJ Banfield LE Gibala MJ Martin Ginis KA. A scoping review of the psychological responses to interval exercise: is interval exercise a viable alternative to traditional exercise? Health Psychol Rev. (2017) 11(4):324–44. 10.1080/17437199.2017.1326011 28460601

[74] Heisz JJ Tejada MGM Paolucci EM Muir C. Enjoyment for high-intensity interval exercise increases during the first six weeks of training: implications for promoting exercise adherence in sedentary adults. PLoS One. (2016) 11(12):e0168534. 10.1371/journal.pone.0168534 27973594 PMC5156428

[75] Kwan BM Bryan AD. Affective response to exercise as a component of exercise motivation: attitudes, norms, self-efficacy, and temporal stability of intentions. Psychol Sport Exerc. (2010) 11(1):71–9. 10.1016/j.psychsport.2009.05.010 20161385 PMC2782828

[76] Teixeira PJ Silva MN Mata J Palmeira AL Markland D. Motivation, self-determination, and long-term weight control. Int J Behav Nutr Phys Act. (2012) 9(1):22. 10.1186/1479-5868-9-22 22385818 PMC3312817

[77] Srismith D Wider L-M Wong HY Zipfel S Thiel A Giel KE Influence of physical activity interventions on body representation: a systematic review. Front Psychiatry. (2020) 11:99. 10.3389/fpsyt.2020.00099 32265747 PMC7096574

[78] WHO. WHO Guidelines on physical activity and sedentary behaviour. Geneva: World Health Organization (2020).

[79] Cairney J McGannon KR Atkinson M. Exercise is medicine: critical considerations in the qualitative research landscape. Qual Res Sport Exerc Health. (2018) 10(4):391–9. 10.1080/2159676X.2018.1476010

[80] Williams TL Hunt ER Papathomas A Smith B. Exercise is medicine? Most of the time for most; but not always for all. Qual Res Sport Exerc Health. (2018) 10(4):441–56. 10.1080/2159676X.2017.1405363

[81] Lind E Ekkekakis P Vazou S. The affective impact of exercise intensity that slightly exceeds the preferred level: ‘pain’ for no additional ‘gain’. J Health Psychol. (2008) 13(4):464–8. 10.1177/1359105308088517 18420754

[82] Ekkekakis P Vallance J Wilson PM Ewing Garber C. Extraordinary claims in the literature on high-intensity interval training (HIIT): III. Critical analysis of four foundational arguments from an interdisciplinary lens. Psychol Sport Exerc. (2023) 66:102399. 10.1016/j.psychsport.2023.102399 37665861

[83] Ekkekakis P Tiller NB. Extraordinary claims in the literature on high-intensity interval training: II. Are the extraordinary claims supported by extraordinary evidence? Kinesiol Rev. (2022) 12(6242):1–14. 10.1123/kr.2022-0003

