# Supplementary material for: “I just had the feeling that the interval training is more beneficial”: young adults' subjective experiences of physical fitness and the role of training modes
Source: Front Sports Act Living. 2023 May 15;5:1115944. doi: 10.3389/fspor.2023.1115944 (PMC10225535; doi:10.3389/fspor.2023.1115944)
Supplement: Supplementary file 1 [file Table1.docx]

Supplementary Material

Supplementary Table 1. Overview of assessed developmental trajectories for the biographical mapping at follow-up II

| Health- and activity-related developmental trajectories |
| --- |
| Amount of PA in everyday life |
| Relevance of sport and exercise |
| Enjoyment of training |
| Unpleasentness of training |
| Motivation to train |
| Health behavior |
| Physical fitness in everday life (i.e. general fitness level) |
| Exercise-related physical fitness (i.e. athletic fitness level or training status) |
| Physical complaints |
| Psychological stress |
| Global well-being |
| Attractiveness |
| Relevance of sport and exercise for social in one’s own social environment |
| Organization of everyday life |
